# Ultrasound-guided preoperative localization of radial nerve in the treatment of extra-articular distal humeral shaft fractures

**DOI:** 10.1186/s12891-021-04954-7

**Published:** 2022-01-03

**Authors:** Weifeng Li, Hui Li, Haiying Wang, Shunyi Wang

**Affiliations:** Department of Orthopaedic Surgery, Baoding No 1 Central Hospital, No. 320, Changcheng Street, Baoding, 071000 Hebei People’s Republic of China

**Keywords:** Ultrasound-guided, Preoperative localization, Radial nerve, Distal humeral shaft fracture

## Abstract

**Background:**

The aim of this study was to discuss the treatment of extra-articular distal humeral shaft fractures using ultrasound-guided preoperative localization of radial nerve.

**Methods:**

Between May 2010 and December 2019, 56 patients with extra-articular distal humeral shaft fractures were retrospectively reviewed. Twenty eight patients were received examination by using preoperative localization of radial nerve guided by ultrasound-guided preoperative localization (group A) and 28 control patients without ultrasound-guided (group B). All patients were treated surgically for distal humeral shaft fractures by posterior approach techniques. Operative time, radial nerve exposure time, intraoperative bleeding volume, union time and iatrogenic radial nerve palsy rate were compared between the two groups. Elbow function was also evaluated using the Mayo Elbow Performance Score (MEPS).

**Results:**

A significant difference was observed between the two groups, Operative time (113.25 min vs 135.86 min) (*P* < 0.001), radial nerve exposure time (20.82 min vs 32.53 min) (*P* < 0.001), intraoperative bleeding volume (246.80 ml vs 335.52 ml) (*P* < 0.001). However, iatrogenic radial nerve palsy rate (3.6% vs 7.1%) (*P* = 0.129), the fracture union time (13.52 months vs 12.96 months) (*P* = 0.796) and the MEPS score (87.56 vs 86.38) (*P* = 0.594) were no significantly different in both groups.

**Conclusions:**

The study demonstrates that ultrasound-guided preoperative localization is an effective approach in the treatment of extra-articular distal humeral shaft fracture by revealing radial nerve, which may help reduce the operative time, radial nerve exposure time and the intraoperative bleeding volume.

## Background

Fractures of the humerus may have a dramatic effect on upper extremity function, and middle and lower humerus fractures account for 1 and 3% of all fractures in adults [1]. The anatomical shape of the distal humerus is special, which is a stress concentration area. Conservative treatment is prone to failure and require open reduction and internal fixation [2]. The intimate relationship of the radial nerve with the shaft of the humerus within the spiral groove makes it particularly vulnerable to traction, transection or entrapment injuries with fractures of the middle and distal third of the humerus [3]. Thus, it is urgent to explore a safe approach that allows accurate exposure. The purpose of this study was to assess the efficiency and convenience of ultrasound-guided localization of the radial nerve in extra-articular distal humeral shaft fracture and to discuss whether ultrasound-guided preoperative localization could help guide surgeons in their operative treatment of distal humeral fractures. The hypothesis was that the ultrasound-guided preoperative localization of the radial nerve may help reduce the operative time and radial nerve exposure time and the intraoperative bleeding volume.

## Material and methods

The study had been approved by the local ethics committee and all patients gave informed consent.

From May 2010 to December 2019, 56 patients with extra-articular distal humeral shaft fractures were operated on using the posterior approach technique. The inclusion criteria were (1) above18 years old, (2) unilateral closed extra-articular distal humeral shaft fracture, and (3) the elbow function were normal pre-operation. The exclusion criteria were: (1) above 60 years old, (2) open or pathological fracture, (3) previous surgery on the injured elbow, (4) preoperative radial nerve or vascular injury, and (5) fracture older than 3 weeks. Twenty eight patients (18 male and 10 female patients; mean age, 39 years; range, 21–58 years) were received examination by using preoperative localization of radial nerve guided by ultrasound-guided preoperative localization (group A) and group B including 28 patients (20 male and 8 female patients; mean age, 36 years; range, 18–56 years) were treated without ultrasound-guided. More demographic characteristics are displayed in Table 1. There was no statistical diference between the two groups.

**Table 1:** Demographic characteristics data of two groups

Characteristic	Group A (*n* = 28)	Group B (*n* = 28)	*P*-value
Gender			
Male	18 (64.29)	20 (71.43)	0.483
Female	10 (35.71)	8 (28.57)	
Age	39.76 ± 8.43	36.63 ± 9.86	0.375
Side			
Right	16 (57.14)	19 (67.86)	0.172
Left	12 (42.85)	9 (32.14)	

Group A, ultrasound-guided group; Group B, without ultrasound-guided group

### Ultrasonography (US) and precise location ultrasound

**Fig. 1:**
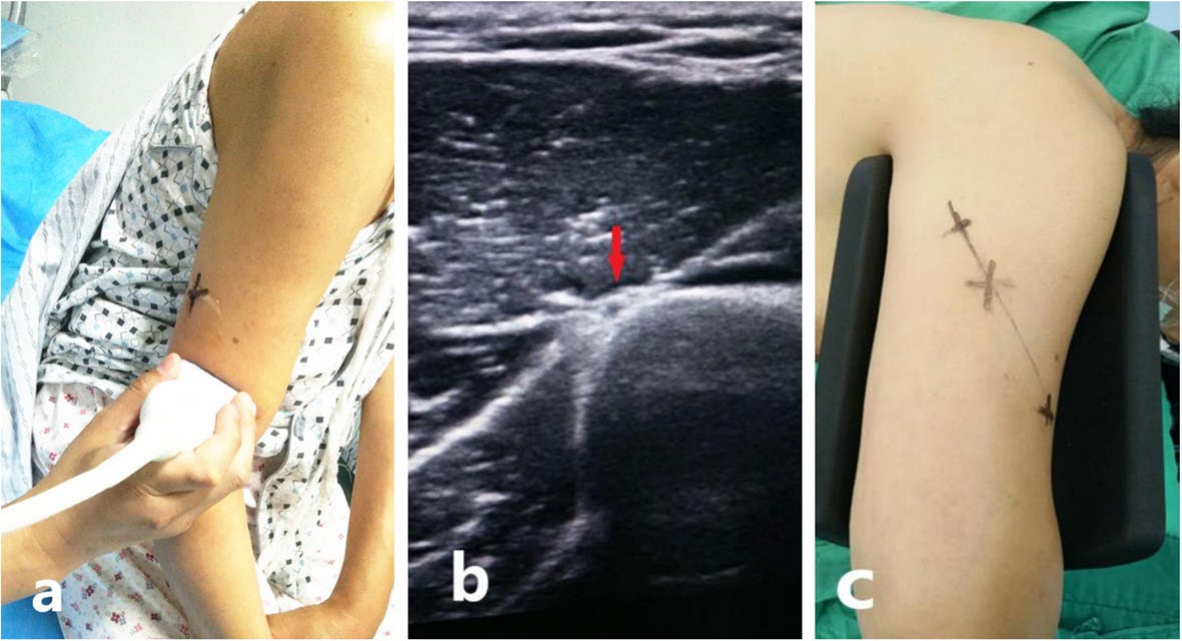
Distal humeral shaft fracture in a 55-year-old female status post bicycle accident. **a** Preoperative localization of radial nerve guided by ultrasound. **b** Ultrasound image at the posterior aspect of the left mid arm shows the intact radial nerve (red arrow). **c** The body surface location of the radial nerve is marked on the skin of the posterior upper arm

All US was performed in the Department of Medical Imaging on Logiq E9 Ultrasound machine (General Electric Healthcare, Chicago, Illinois, USA) with 6–15 MHz high-resolution multifrequency linear transducer by a trained musculoskeletal sonographer. The patient’s discomfort should be adequately assessed prior to the ultrasound examination and regional block are required for patients with severe pain. The position of the radial nerve was demarcated by ultrasound technologist through preoperative ultrasound examination, which was marked on the skin of the posterior upper arm (Fig. 1).

### Surgical technique

**Fig. 2:**
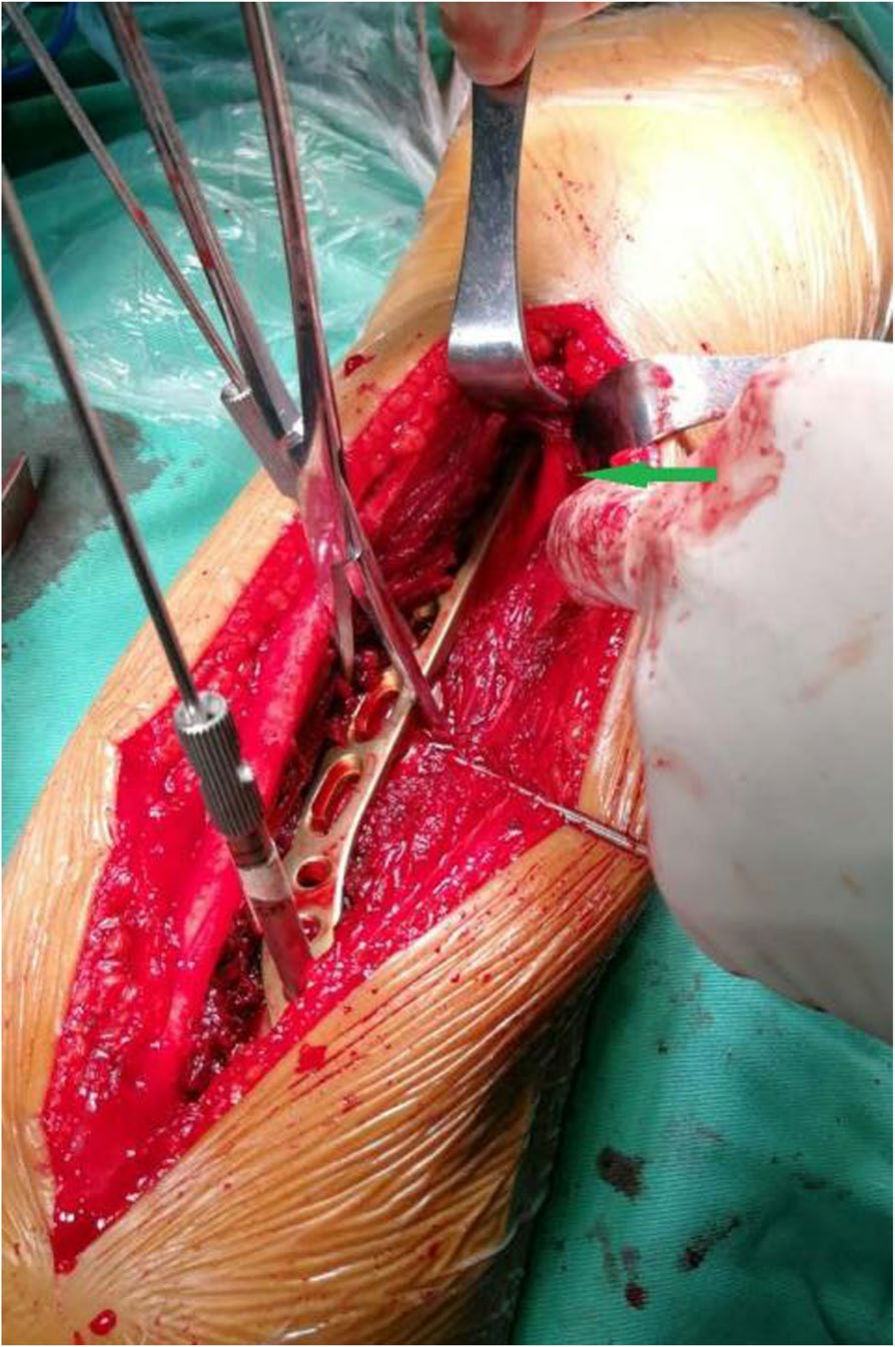
Intraoperative photograph of the posterior upper arm shows normal radial nerve (green arrow)

A posterior approach was applied to all cases. A skin incision was performed following a line from the olecranon to the proximal third of the posterior arm. Subsequently, a approach by splitting the triceps belly along its fibers was used to expose the posterior humeral shaft. Radial nerve was carefully dissected and fully released following the spiral groove (Fig. 2), and fracture reduction clamps were used to reduce the fracture fragments. An extra-articular distal humeral plate was applied centrally over the posterior surface of humeral shaft and locking screws were placed on the either side of the humeral fracture to stabilize the reduction [4]. Postoperative treatment and effect evaluation flexion–extension exercises were executed in both groups at 3 days post operation. The operative time, radial nerve exposure time, intraoperative bleeding volume and iatrogenic radial nerve palsy rate were recorded. Clinical follow-ups and radiological evolutions were obtained regularly each month. Post-operative follow-up for 1 year, the function of the elbow was assessed by the MEPS [5–7]. The scale considered a result of > 90 points as excellent, 75–89 points as good, 60–74 points as acceptable and < 60 points as poor.

### Statistical analysis

SPSS statistical software (version 22.0; SPSS, IL, USA) was performed for data analyses. All data were presented as the mean and standard deviation. The categorical values was used by the Chi-squared test. After establishing data normality, and independent sample t test was used to evaluate the differences between the two groups. The categorical values were analyzed using Chi-squared test. *P*-value < 0.05 was defined as the threshold for statistical significance.

## Results

The mean follow-up time was 16 months (13–24 months). The demographic characteristics showed no significant differences in age, gender and side between the two groups (Table 1).

**Fig. 3:**
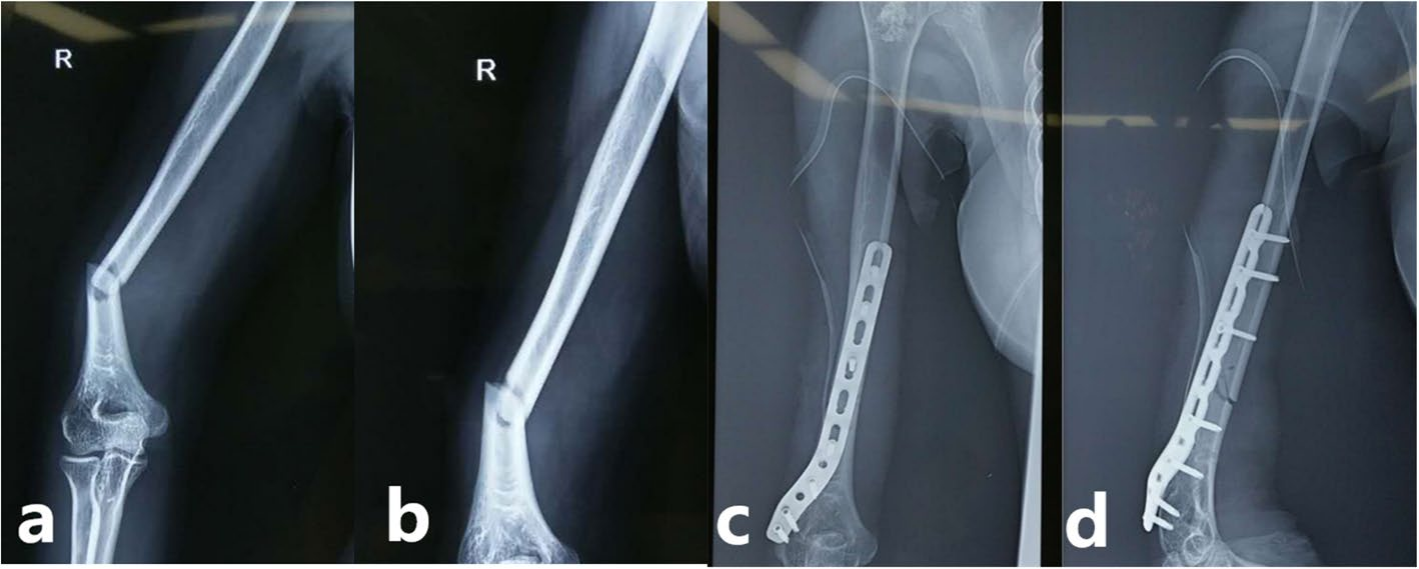
Normal radial nerve in a 55-year-old female with distal humeral shaft fracture following a bicycle accident. **a** Anteroposterior and **b** lateral preoperative radiographs of the right humerus shows moderately displaced and oblique distal humeral shaft fracture. **c** Anteroposterior and **d** lateral postoperative radiographs of the right humerus obtained 2 days later show a distal humeral plate with 7 cortical screws transfixing the right distal humeral shaft fracture in anatomic alignment and without hardware complication

Many measurements showed significant differences between the two groups (Table 2). The group A showed shorter operative time (113.25 min vs 135.86 min) (*P* < 0.001), shorter radial nerve exposure time (20.82 min vs 32.53 min) (*P* < 0.001), and less intraoperative bleeding volume (246.80 ml vs 335.52 ml) (*P* < 0.001) than in Group B. However, There was not significant difference in the iatrogenic radial nerve palsy rate between the two groups (3.6% vs 7.1%) (*P* = 0.129). When comparing fracture union time, both groups showed no significant difference (13.52 months vs 12.96 months) (*P* = 0.796). There was no significant difference in the MEPS score between the two groups (87.56 vs 86.38, *P* = 0.594) (Table 3). There was no failure of internal fixation in either group (Fig. 3). One superficial wound infection occurred, which, did not require any special treatment.

**Table 2:** Comparison of operation time, radial nerve exposure time and intraoperative bleeding volume between two groups

Indexes	Group A	Group B	*P*-value
Operative time (min)	113.25 ± 15.92	135.86 ± 17.46	< 0.001
Radial nerve exposure time (min)	20.82 ± 5.53	32.53 ± 15.88	< 0.001
Intraoperative bleeding volume (ml)	246.80 ± 16.26	335.52 ± 14.37	< 0.001
Iatrogenic radial nerve palsy rate (%)	3.6	7.1	0.129

Group A, ultrasound-guided group; Group B, without ultrasound-guided group

**Table 3:** Comparison of clinical outcomes between two groups

Indexes	Group A	Group B	*P*-value
Fracture union time (months)	13.52 ± 3.9	12.96 ± 3.4	0.796
MEPS (points)	87.56 ± 7.53	86.38 ± 7.82	0.594

Group A, ultrasound-guided group; Group B, without ultrasound-guided group

## Discussion

The distal humerus is the junction of cylinder and triangle which is weak spot of humerus mechanics. The local anatomy is irregular, especially the metaphysis which often leads to fracture after being subjected to violence [8–10]. Fracture is common transverse, spiral and comminuted, more affect the elbow joint function. Conservative treatment is easy to fail. Open reduction and internal fixation should be the main treatment of choice in case of middle and distal humeral fractures associated with radial nerve palsy or not [11–13]. The classical surgical approach is the lateral approach, which allows direct exposure of the radial nerve and supine patient position. However, the posterior antebrachial cutaneous nerve could be at the risk of iatrogenic injury [14]. In recent years, lateral anatomic plate has been widely used in this fracture [15, 16]. Certainly, the posterior approach offers undoubted advantages in terms of exposure of the fracture and visualization of the radial nerve [11]. In this study, we used a posterior approaches in the management of extra-articular distal humeral shaft fracture. The radial nerve resides in the spiral groove 15 cm proximal to the humeral articular surface and runs between the brachioradialis and the brachialis muscles. Radial nerve contusion or complete fracture is more likely to occur due to pulling and clamping at the fracture. Iatrogenic nerve injuries are well known in the medical literature and orthopedic surgery [13, 17, 18]. The most frequent cause of iatrogenic nerve injury is interruption of the nerve continuity during surgery or medical procedure [19]. Zhao et al. [20, 21] emphasized that precision for the exploration of the radial nerve was essential to avoid nerve iatrogenic injuries. How to achieve radial nerve exposure remains a challenge for the orthopaedic surgeon. It is crucial to choose ultrasound-guided examination for accurate location of radial nerve.

During the past decade, US has become an important diagnostic tool in musculoskeletal radiology. US for peripheral nerve diagnosis has gained popularity due to its cost-effectiveness and non-invasive nature with very low risk [22]. It can be used to demonstrate radial nerve with respect to position, swelling, loss of continuity and partial laceration. In the study, the precise location of the radial nerve was the key step, and the position of the radial nerve through the spiral groove was demarcated by the authors through preoperative ultrasound examination, which was marked on the skin of the posterior upper arm. The radial nerve could be quickly located and protected during the operation, according to a well marked label. In the present study, the mean operative time was shorter in the group A (113.25 min) than in group B (135.86 min) (*P* < 0.001). Significant difference was seen such as the mean intraoperative bleeding volume (group A, 246.80 ml; group B, 335.52 ml; *P* < 0.001). The mean radial nerve exposure time showed the greater difference: 20.82 min in group A versus 32.53 min in group B (*P* < 0.001). Shorter exposure times for the radial nerve could reduce intraoperative bleeding, lower subsequent infection, enhance surgeon confidence and reduce iatrogenic injury of the radial nerve. In our study, the incidence of iatrogenic radial nerve palsies was 3.6% (1/28) in group A, which is lower than 7.1% (2/28) in group B, which was no significant difference between the two groups. Despite careful radial nerve visualization procedures, there were still three cases of radial nerve palsy in the two groups. In the study, we have found that through ultrasound-guided radial nerve exploration, the posterior approach allows for optimal management of complex and multi-fragmentary fractures.

Operation skills and notes. (1) Preoperative localization should be performed gently and requires highly skilled Ultrasonographer to quickly locate the radial nerve. Our experience is as follows. The radial nerve closest to the cortical bone was marked as central point 1; the points 2 and 3 were determined at the position of the radial nerve 3–5 cm above and below the point 1; the line connecting 123 points was the approximate location of the radial nerve. (2) The patient should be kept in a lateral position with the arm drooped freely on a cylindric arm board which allows the elbow to be bended to 90 degrees. (3) During the operation, the radial nerve in a tension-free state needs to be labeled with a rubber tissue, which contributes to the protection and exposure of the radial nerve. We used the a posterior approach in the secure zone (2 cm away from of the radial nerve), therefore, skin and triceps could be quickly separated. The marked area of the radial nerve should be carefully separated, and location of the radial nerve could be confirmed by palpation with the surgeon’s index finger.

This study still has several limitations. First, Ultrasound technology cannot be mastered by some surgeons. Ultrasound examinations are performer dependent and require an experience in soft tissue structures which has a relatively long learning curve. Second, preoperative operation may aggravate the patient’s pain and discomfort. Third, the follow-up sample size was insufficient and additional studies with larger sample numbers are needed to enhance the credibility of the conclusion.

## Conclusions

In conclusion, the study showed that ultrasound-guided preoperative localization is an effective approach in the treatment of extra-articular distal humeral shaft fracture by revealing radial nerve, which may help reduce the operative time, radial nerve exposure time and the intraoperative bleeding volume. In addition, it has the advantages of operability, safety, efficiency and repeatability.

## Data Availability

The detailed data and materials of this study were available from the corresponding author through emails on reasonable request.

## Abbreviations

MEPS: Mayo Elbow Performance Score
US: Ultrasonography
